# Pterygium—The Good, the Bad, and the Ugly

**DOI:** 10.3390/cells10071567

**Published:** 2021-06-22

**Authors:** Sara I. Van Acker, Bert Van den Bogerd, Michel Haagdorens, Vasiliki Siozopoulou, Sorcha Ní Dhubhghaill, Isabel Pintelon, Carina Koppen

**Affiliations:** 1 Antwerp Research Group for Ocular Science (ARGOS), Translational Neurosciences, Faculty of Medicine, University of Antwerp, 2610 Wilrijk, Belgium; bert.vandenbogerd@uantwerpen.be (B.V.d.B.); michelhaagdorens@gmail.com (M.H.); nidhubhs@gmail.com (S.N.D.); carina.koppen@uza.be (C.K.); 2 Department of Pathology, Antwerp University Hospital, University of Antwerp, 2650 Edegem, Belgium; vasiliki.siozopoulou@uza.be; 3 Center for Oncological Research (CORE), Integrated Personalized and Precision Oncology Network (IPPON), University of Antwerp, 2610 Wilrijk, Belgium; 4 Department of Ophthalmology, Antwerp University Hospital, 2650 Edegem, Belgium; 5 Laboratory of Cell Biology and Histology, Faculty of Pharmaceutical, Biomedical and Veterinary Sciences, University of Antwerp, 2610 Wilrijk, Belgium; isabel.pintelon@uantwerpen.be

**Keywords:** pterygium, ocular surface squamous neoplasia, skin cancer, ultraviolet radiation, atypia, dysplasia, preneoplasia

## Abstract

Pterygium is a multifaceted pathology that displays apparent conflicting characteristics: benign (e.g., self-limiting and superficial), bad (e.g., proliferative and potentially recurrent) and ugly (e.g., signs of preneoplastic transformation). The natural successive question is: why are we lacking reports showing that pterygium lesions become life-threatening through metastasis, especially since pterygium has considerable similarities with UV-related malignancies on the molecular level? In this review, we consider how our pathophysiological understanding of the benign pterygium pathology overlaps with ocular surface squamous neoplasia and skin cancer. The three UV-related disorders share the same initial insult (i.e., UV radiation) and responsive repair mechanisms to the ensuing (in)direct DNA damage. Their downstream apoptotic regulators and other cellular adaptations are remarkably alike. However, a complicating factor in understanding the fine line between the self-limiting nature of pterygium and the malignant transformation in other UV-related diseases is the prominent ambiguity in the pathological evaluation of pterygium biopsies. Features of preneoplastic transformation (i.e., dysplasia) are used to define normal cellular reactions (i.e., atypia and metaplasia) and vice versa. A uniform grading system could help in unraveling the true nature of this ancient disease and potentially help in identifying the earliest intervention point possible regarding the cellular switch that drives a cell’s fate towards cancer.

## 1. Introduction

**Figure 1:**
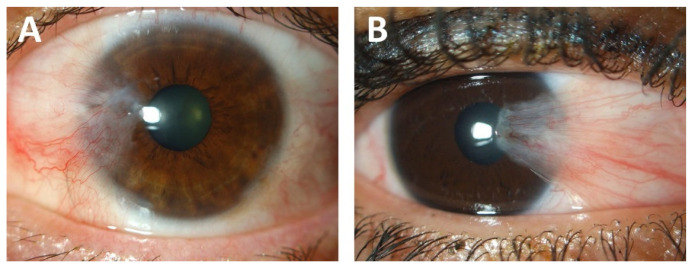
Clinical representation of grade III (**A**) and grade IV (**B**) pterygium. Ocular surface photographs were taken at the Antwerp University Hospital and graded according to the Johnston, Williams, and Sheppard classification system [7].

Pterygium is a disease that dates back to antiquity. The name itself is derived from the ancient Greek ‘pterygos’, or wing, and the first clinical descriptions date back to 1000 B.C.E. [1]. Despite centuries of experience, fundamental questions still exist regarding the aetiology and nature of this common lesion. Clinically, pterygium appears as a wing-shaped conjunctival thickening that migrates over the corneal limbus, typically from the nasal side, which can encroach and cover the central cornea and visual axis (Figure 1). While the development of pterygium may be slow, recurrence after excision occurs frequent and rapidly in the absence of meticulous surgical resection [2]. It is important to consider why an ostensibly benign lesion displays such a local proliferative and aggressive phenotype.

The pathogenesis of pterygium remains elusive. It has been described as a degenerative disorder due to the prominent elastotic degeneration of the stroma [3], and as a localized region of stem cell deficiency. It has also been suggested that it could be a premalignant condition based on histology [4,5,6]. The suggestion that it could be premalignant is worth examining as, clinically, this does not appear to be the case. In this report, we present common risk factors, pathways of damage induction, cytological and histological features of pterygium and compare them to typical preneoplastic characteristics. We then consider why pterygium is ultimately a local non-cancerous entity despite bearing so many similarities to epidermal cancers and ocular surface squamous neoplasia (OSSN).

## 2. Risk Factors for Pterygium Development

**Figure 2:**
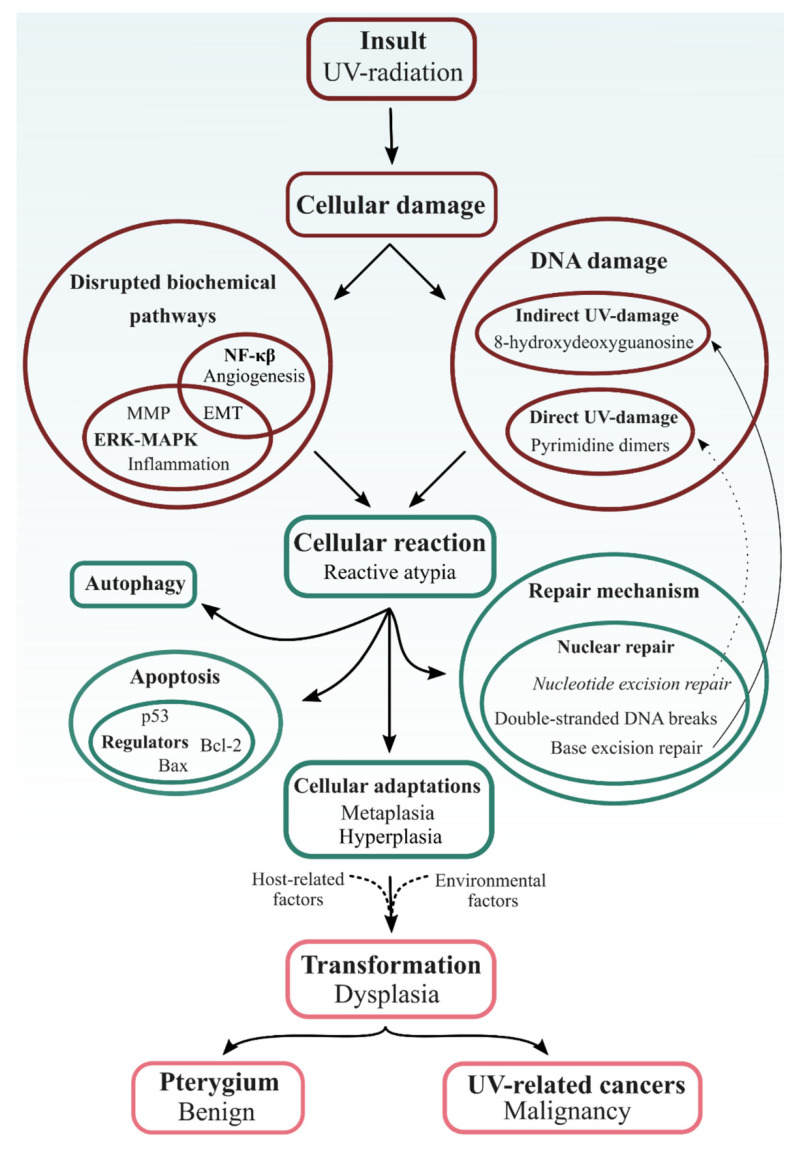
Schematic representation of the similarities between pterygium, ocular surface squamous neoplasia and epidermal cancer, starting from the initial insult (i.e., UV radiation) to the damage shared between the UV-related lesions and their uniform cellular reactions. The difference between pterygium and true malignant cancers lies within the final step. EMT, epithelial-to-mesenchymal transition; ERK-MAPK, extracellular-signal-regulated kinase–mitogen-activated protein kinase; MMP, matrix metalloproteinases.

A wide range of intrinsic and extrinsic factors can cause cellular damage throughout one’s life. Although their triggers are considerably diverse, they all disrupt cellular homeostasis by one or more of four primary biochemical mechanisms: (I) ATP depletion, (II) permeabilization of cell membranes, (III) disruption of biochemical pathways and (IV) DNA damage [8]. When examining the extensive epidemiological data on pterygium, it is clear that ultraviolet (UV) radiation is the key physical detrimental factor (Figure 2) [9]. The region 37° to the north and south of the equator was even named the pterygium belt in 1965 [10]. Hence, pterygium—along with pinguecula, climatic keratopathy, actinic granuloma, ocular surface and eyelid malignancies, cataract, etc.—has been classified as an ‘ophthalmoheliose’; i.e., a pathogenesis in which UV radiation has been implicated, with varying degrees of certainty [11]. To re-establish cellular homeostasis and withstand UV-induced stress, the exposed tissue exploits several mechanisms. These include the induction of cellular repair pathways, the activation of cellular adaptations (such as hyperplasia and metaplasia), the promotion of autophagy and the initiation of cell death (Figure 2) [12,13].

As all cells contain general basic mechanisms to regain homeostasis after insults, it is reasonable to assume that there is a common pathway linking pterygium to other proliferative UV-related diseases, such as epidermal cancer and OSSN. Using epidermal cancer as an example, UV radiation contributes to its development by interacting with signal transduction pathways (e.g., protein kinase C signaling and the c-Jun N-terminal kinase pathway), leading to an altered gene expression pattern (Figure 2) [14]. Furthermore, UV affects the integrity of DNA both directly and indirectly through oxidative stress-induced cascades. Such unrepaired DNA damage leads to mutagenesis [14]. Both mechanisms also play a significant role in pterygium initiation and formation (Figure 2) [15,16,17,18,19]. Yet, pterygium only slowly evolves and remains superficial, while UV-related cancers can metastasize and become life-threatening.

## 3. UV-Induced Damage

### 3.1. Biochemical Pathway Disruption

The impact of one specific UV-activated signaling pathway is already well-established in pterygium—the extracellular-signal-regulated kinase (ERK), a mitogen-activated protein kinase (MAPK) pathway. This pathway connects signals from the extracellular milieu with the intracellular machinery, thereby controlling several fundamental processes such as proliferation, differentiation, apoptosis and migration [20]. Due to its broad influence on cellular fate, it is not surprising that one-third of all human cancers are characterized by a dysregulated ERK pathway, including non-melanoma skin cancer [20,21]. Despite an established role in one-third of all cancers and pterygium, it is currently unclear whether this is also the case for UV-related ocular lesions. ERK signaling has only been demonstrated to contribute towards a hyperproliferative cellular status in an OSSN mouse model [22].

UV radiation is known to be involved in the ligand-independent autophosphorylation of the epidermal growth factor receptor, thereby triggering the ERK pathway in pterygium [15,16]. The activation of this pathway contributes to the expression of prominent players, such as matrix metalloproteinase-1 (MMP-1), interleukin (IL)-6, IL-8 and vascular endothelial growth factor (VEGF) [15,16,17]. MMP-1 is one of six MMPs found in the limbal basal cells affected by pterygium, which are also present in invasive tumors [23]. As MMP-1 is localized in pterygium-diseased cells that invade the cornea, it is hypothesized that MMP-1 is involved in the pathogenesis by facilitating Bowman’s membrane dissolvement, thereby enabling the migration and local infiltration of pterygial cells [23]. Furthermore, tear films of patients surgically treated for pterygium show a clear change and decrease in VEGF and IL-6/8 levels one year after surgery, emphasizing their role in pterygium pathology [24].

Despite being less described than the ERK-MAPK pathway, it seems that the aberrant activation of the NF-κβ pathway also contributes to the alteration of signaling switches associated with both pterygium and tumorigenic processes. Starting with the original UV radiation trigger, it is known that the 280–320 nm wavelength range (i.e., predominantly UVB spectrum) is able to stimulate the NF-κβ pathway in keratocytes and ocular surface epithelia [25,26]. The corresponding influence of its activation is considerably comprehensive, as NF-κβ targets genes that intervene in proliferation, apoptosis, angiogenesis and epithelial-to-mesenchymal transition (EMT) [27]. It is therefore not surprising that NF-κβ has an active role in cancer initiation and development, including the maintenance of cancer stem-like cells [28] and metastasis [27]. The NF-κβ pathway and related cytokines are also believed to represent a cross talk route between pterygium and dry eye disease [29].

When examining pterygium, evidence of the NF-κβ signaling relies on the increased levels of phosphorylated NF-κβ inhibitors in the cytoplasm and the expression of NF-κβ-related genes [30,31,32]. The link between an active NF-κβ pathway and the resulting angiogenesis and EMT is of importance, as both events are linked to a malignant transformation. Active angiogenesis has, for example, been demonstrated in premalignant lesions of cutaneous squamous cell carcinoma, indicating an early event in its development. Angiogenesis further accelerates at each disease stage [33]. Furthermore, EMT increases the malignant character of lesions as it enhances their invasiveness and metastatic activity [34]. The migratory capability of pterygium cells is believed to be enhanced through EMT as well, and it is grounded in its histopathological and molecular identification in surgically excised pterygia [35]. The role of EMT signaling in primary and recurrent pterygium is still being explored [36,37,38,39]; however, both the ERK-MAPK and NF-κβ pathways are recognized as engaged [36,37].

Several studies have already described (lymph)angiogenesis as a part of pterygium pathology [40,41,42]. The pathological contribution of the NF-κβ pathway has been established, as its downregulation in pterygium cells results in a significant decrease in the expression of one of the angiogenic key contributors, i.e., VEGF [43]. However, the exact role of VEGF in pterygium pathology needs to be further elucidated. Liu et al., found significantly elevated tear film levels of VEGF in progressive pterygium compared to inactive pterygium, but failed to find a significant difference in tear film concentrations in pterygium patients compared to healthy individuals [29]. The latter confirms our previous results and strengthens our hypothesis that interindividual variability could play an important role, as the VEGF levels are considered elevated with respect to patient-specific healthy values [24]. On the contrary, Uthaithammarat et al., did demonstrate higher VEGF concentrations in pterygium patients compared to the healthy control group [44]. Although the exact players and their corresponding contributions still need to be defined and/or further refined, it is important to note that there is a difference in the organization of the vascular network when it belongs to pterygium or a malignant transformed lesion. Tumor angiogenesis occurs in an uneven, chaotic and serpentine manner that is characterized by irregular branches and arterio-venous shunts [45]. On the other hand, despite the fact that vessel density is greatly increased in pterygium, the vessels are less tortuous and even more organized when compared to a healthy conjunctiva [42]. As these observations were made in early stage primary pterygium [42], it would be interesting to know whether the vasculature resembles tumor angiogenesis in more advanced stages or in recurrent pterygium.

### 3.2. DNA Damage

The nuclear content of pterygial cells also shows signs of direct and indirect damage by UV radiation. Upon exposure to UV-light, pyrimidine dimers commonly arise in DNA strands, facilitating mutagenesis [14]. The predominant cytosine to thymine base substitution at pyrimidine sites is unique to UV-insults, and is therefore known as the ‘UV-signature’ [46]. OSSN and squamous cell carcinoma of the skin exhibit this molecular signature, especially in the *TP53* gene [46,47,48]. One study defined the mutation spectrum of the *TP53* gene in pterygium, and one out of six missense mutations encompassed a cytosine to thymine transversion (exon 5, codon 179, CAT) [49]. As codon 178 of exon 5 consists of a CAC combination, we can conclude that this substitution arise at a pyrimidine site. Nevertheless, another type of pyrimidine dimers, i.e., thymine dimers, has been found abundantly in pterygium, notably in the epithelial, stromal and even vascular compartment [18]. The presence of thymine dimers is also considered as a hallmark of UV-induced DNA damage and is characterized by a complex mutation spectrum [50,51]. Furthermore, UV is capable of inducing DNA damage indirectly through the formation of free radicals, i.e., superoxide anions, hydrogen peroxide and peroxynitrite, that lead to oxidative stress [52]. Entering the nucleus, these agents form DNA 8-hydroxydeoxyguanosine in UV-exposed epidermis in a corneal mouse model and in pterygium patients [19,53,54]. DNA 8-hydroxydeoxyguanosine is highly mutagenic, promoting guanine to thymine transversion [55].

## 4. UV-Induced Cellular Reactions

### 4.1. Nuclear Repair Mechanisms

In health, DNA damage at the ocular surface is restored by nucleotide and base excision repair mechanisms [14,56,57]. The crucial role of these protection mechanisms is particularly evident in patients with xeroderma pigmentosum (XP). These patients are hypersensitive to UV-light due to genetic alterations in genes responsible for DNA repair [58]. The lack of DNA repair after UV radiation results in a 10,000-fold increased risk of developing basal and squamous cell carcinomas [59]. Furthermore, both pterygium and ocular neoplasia formation are frequently reported in XP patients, showing a fourfold to 100,000-fold incidence increase, respectively [60,61,62,63,64,65]. While the exact involvement of nuclear excision repair in pterygium initiation has not yet been explored, the accumulation of the highly mutagenic 8-hydroxydeoxyguanosine in pterygial cells can induce the expression of 8-oxyguanine glycosylase, the enzyme responsible for its removal [19]. The importance of this base excision repair mechanism is further emphasized by genetic polymorphisms being identified in the *OGG1* gene, as well as in the *XRCC1* gene, which renders patients more prone to developing pterygium [66,67].

Alongside the aforementioned DNA damage, UV radiation itself can cause dsDNA breaks [68]. If not repaired correctly, they can lead to deletions, translocation and fusions in the DNA [69]. When focusing on pterygium, the relevance of repairing dsDNA breaks is seen in polymorphisms in corresponding repair genes that are associated with a genetic predisposition to pterygium. The T-991C polymorphism in the promotor of the *XRCC6* gene, for example, is active in nonhomologous end-joining repair of dsDNA-breaks [70]. Furthermore, expression patterns of the homologous repair genes *RAD50* and *RAD54* seem altered as increased and decreased levels are found in pterygial tissue compared to unaffected tissue, respectively [71]. The analogy continues, as similar polymorphisms linked to epidermal cancer susceptibility are also involved in other nonhomologous end-joining repair (i.e., *LIG4*) and homologous recombination (i.e., *XRCC2* and *XRCC3*) genes [72].

### 4.2. Autophagy

Autophagy is a self-digesting cellular process responsible for removing long-lived proteins, damaged organelles and malformed proteins through a strictly regulated lysosomal pathway [73]. This degradation pathway maintains homeostasis and thereby orchestrates growth, differentiation, response to oxidative stress and nutrient deficit, macromolecule and organelle turnover and, finally, cell death [73]. Autophagy is, however, a complex process that plays a context-dependent role in UV response, harboring both tumor-promoting and tumor-suppressing characteristics [74]. Similarly, during tumor development and at the tumor–immune interface, autophagy can be a suppressor or driver of tumorigenesis, depending on the features of the heterogenous and multifaced tumor microenvironment [75,76,77]. While only one report has described a considerable inhibition so far [78], it is likely that disturbances in normal autophagic processes are involved in the inappropriate proliferative capacity of pterygium.

### 4.3. Apoptosis

While less studied than DNA damage, altered expression patterns of the apoptotic regulators p53, Bcl-2 and Bax have been shown in pterygium [79,80]. To emphasize the role of programmed cell death as cellular reaction, we take p53 by way of an example. The *TP53* suppressor gene is a well-characterized protector against UV-induced carcinogenesis, both in the epidermis and at the ocular surface [81]. Depending on the amount of DNA damage, p53 allows the cell to repair the UV-induced damage through cell cycle arrest or activates the apoptosis pathway [82]. Unfortunately, *TP53* mutations occur early in the course of UV-irradiation and represent a corresponding early event in carcinogenesis [81,83]. The consequent inactivation of the *TP53* tumor suppressor gene results in an unretained cell proliferation, impeded cell death and genomic instability [84].

As discussed previously, mutations in the *TP53* gene can be found in UV-related lesions. The *TP53* mutation database of the International Agency for Research on Cancer (Version R20, https://p53.iarc.fr, accessed on 4 January 2021) reports more than 29,900 somatic mutations, which can lead to an aberrant or abolished protein production. Early on, it was discovered that missense mutations in the *TP53* DNA-binding core cause the predominant loss of function [85]. Mutant p53 proteins have a prolonged half-life, the extension of which is mutation-dependent [86]. Such accumulation enables pathologists to identify p53-affected cells immunohistologically and for it to be proposed as an adjunct to the routine diagnosis of preneoplastic lesions, especially in questionable cases [87,88]. Considering that *TP53* mutations are an early sign of carcinogenesis, it is remarkable that several studies report p53 positivity in pterygium [89]. However, p53 positivity still needs to be cautiously interpreted, as it perfectly demonstrates the dual character of pterygium. Mutations in the *TP53* gene can correlate with the protein levels, as deletion mutations corresponding with a stop codon or truncated proteins and missense mutations indeed result in negative and positive p53 staining, respectively [49]. This correlation did, however, not apply to all specimens [49,90]. Hence, an early indication of carcinogenesis is one of the possible interpretations of a positive p53 staining in pterygium. Another explanation for the suppressed p53 transcriptional activity, despite abundant p53 levels, can be attributed to the actions of an inhibitory p53 binding protein known as mouse double minute 2 (MDM2). This protein is responsible for the p53 translocation from the nucleus to the cytoplasm and its consequent degradation through ubiquitination [91]. Through the administration of an MDM2 antagonist, transcriptional activity could be restored in primary pterygium cells and the viability was consequently reduced [92,93]. Lastly, it is remarkable that the ‘hot spot’ of the *TP53* gene mutations in pterygium correspond to the ‘hot spot’ of malignant melanoma rather than cutaneous squamous and basal cell carcinoma [49].

### 4.4. Cellular Adaptations

**Figure 3:**
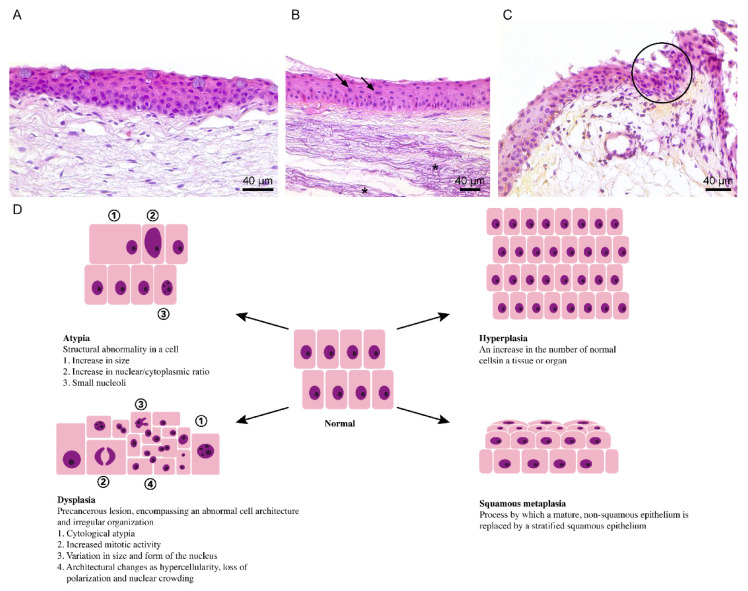
Illustrative hematoxylin and eosin staining of (**A**) a healthy conjunctiva, (**B**) reactive atypia (black arrow) and solar elastosis (asterisk) in pterygium and (**C**) low-graded dysplasia (blue circle) in pterygium, combined with (**D**) a schematic representation of atypia, hyperplasia, squamous metaplasia and dysplasia. Reactive atypia is identified based on the presence of an aberrant cellular morphology, an enhanced nuclear-to-cytoplasmic ratio and a clear nucleolus (**B**). Dysplasia is recognized due to the presence of hyperchromasia, nuclear crowding and hypercellularity (**C**). To aid in distinguishing the cellular adaptations and transformation from one another, definitions and characteristic features are included in the schematic representation (**D**).

When cells are exposed to repetitive or continuous stress, they respond through various cellular adaptative reactions: hypertrophy, hyperplasia, metaplasia and atrophy [8]. However, based on the complex interaction between the exposure pattern and other factors such as gender, age, genetic background and lifestyle, an individual can develop preneoplastic lesions (i.e., dysplasia) or even malignancies (Figure 2) [94]. In oral squamous cell carcinoma, hyperplasia progresses over an increasing degree of dysplasia towards carcinoma in response to either continued tobacco exposure, chronic inflammation, alcohol and/or viral infections [95,96]. Similarly, a metaplasia-dysplasia-cancer sequence occurs in the lung airway, cervix, stomach and pancreas [97]. The most likely adaptive mechanism to chronic UV radiation is hyperplasia, as it protects the epidermis against UV-penetration (Figure 3D) [98]. Indeed, the presence of hyperplasia, dysplasia and squamous cell carcinoma has been shown in mouse epidermis following a long-term UV radiation period [99].

Routine hematoxylin and eosin staining of resected pterygia shows clear signs of reactive atypia (Figure 3B), solar elastosis (Figure 3B), dysplasia (Figure 3C) and inflammation. The identification of solar elastosis, i.e., degeneration of elastic tissue due to UV-irradiation, is not surprising, as prolonged sun exposure is one of the principal risk factors for developing pterygium. Pathologists define reactive atypia based on an increased cellular size, alterations in nuclear-to-cytoplasmic ratio and the presence of small nucleoli (Figure 3B,D). Dysplasia is identified through cytological atypia, along with signs of mitosis, nuclear polymorphisms and architectural changes such as hypercellularity, loss of polarization and nuclear crowding (Figure 3C,D). Both epithelial and goblet cell hyperplasia have also been identified in pterygium [4,100,101,102]. To evaluate the staining correctly, it is important to keep in mind that the ocular surface is continuously exposed to environmental challenges. Isolated reactive atypia can therefore occasionally be found in clinically normal conjunctiva [100].

The presence of squamous metaplasia is, however, a problematic area, as the classical pathological knowledge conflicts with the current pterygium state-of-the-art. Multiple reports uniformly describe squamous metaplasia in pterygium [102,103,104,105,106,107,108,109,110]. Squamous metaplasia is known as the process by which a mature, non-squamous epithelium is replaced by a stratified, squamous epithelium (Figure 3D) [111]. Based on this definition, squamous metaplasia cannot take place in pterygium, as the epithelium of the bulbar conjunctiva is already a squamous epithelium (Figure 3A) [112]. Pterygium biopsies have also been ascribed with squamous metaplasia without well-defined criteria, adding to the confusion [10]. It is therefore clear that different definitions of metaplasia are at the root of this ambiguity. The ‘metaplastic’ transformation is defined using three different methods: grading systems for impression cytology, morphological characteristics and cellular processes.

Grading systems for impression cytology—Four different systems have been published to evaluate the severity of metaplasia in impression cytology samples of pterygium: Murube and Rivas’s grading system [103,104], Nelson’s classification [106], Tseng’s grading system [110] and Wittpen’s grading system [109]. The criteria used for assessment are related to the nuclear/cellular morphology, epithelial cell size, nucleus-to-cytoplasmic ratio, cytoplasmic staining characteristics, goblet cell density, cellular organization and keratinization. These characteristics are, however, not squamous metaplasia-specific, as they are also used to define atypia (see above) and dysplasia (see above and below).

Morphological characteristics—Despite the availability of aforementioned grading systems, some researchers only use a combination of morphological parameters to define squamous metaplasia in epithelial impression cytology samples: goblet cell density [108], nucleus-cytoplasmic ratio [102,108], cell size and shape (i.e., cellular enlargement and elongation) [102,108] and morphological nuclear changes [102,108]. Again, these (combinations of) morphological criteria are not characteristic solely for squamous metaplasia.

Cellular processes—Li et al. identified squamous metaplasia in resected pterygium tissue itself [107], and provided molecular evidence for two criteria, i.e., hyperproliferation and abnormal differentiation [107]. Firstly, hyperproliferation is assessed through the expression of p63, a protein involved in both maintaining cell proliferation in basal progenitor cells and initiating stratification and differentiation [113]. A strong p63 expression has also been demonstrated throughout the pterygium epithelium, confirming the presence of metaplasia based on the first criteria [107]. Secondly, abnormal differentiation was seen through changes in cytokeratin (CK) pairs, given that each cell type within a particular epithelial tissue has a unique profile of CK pairs, isoforms and modification state [114]. Terminal keratinocyte differentiation and keratinization are characterized with a distinctive expression of CK1, and subsequently CK10 [115]. As anticipated, CK10 cannot be detected throughout the nonkeratinized corneal, limbal and conjunctival epithelium, while the superficial layers in pterygium samples are indeed CK10-positive, indicating abnormal differentiation [107,116]. Interestingly, the change in differentiation pattern elucidated by abnormal CK10 expression may also occur without its association with the term ‘squamous metaplasia’ [117]. It could be argued that the first and second criteria are more accurate for describing hyperplasia and keratinization, respectively.

## 5. Dysplasia and Ocular Surface Squamous Neoplasia

Despite the fact that pterygium behaves clinically as a non-cancerous lesion, signs of dysplasia can be demonstrated in surgically resected pterygium (Figure 3C). The terminology and basic characteristics of dysplasia have remained unchanged since 1987 [118]. Dysplasia is defined as a precancerous lesion, encompassing an abnormal cellular architecture along with its irregular organization within a tissue. Grossniklaus et al., described a severity spectrum ranging from mild to severe [118]. In the case of mild dysplasia, signs of hyperchromasia, pleomorphism and loss of cell polarity can be found in one-third of the epithelium. The same cytological features are found in moderate dysplasia; however, they involve three quarters of the epithelium thickness. When only the top epithelial layer is spared, the lesion is defined as severe dysplasia. As soon as the normal surface layer of epithelial cells disappears, the affected tissue is categorized as carcinoma in situ. The following state of squamous cell carcinoma is reached when the dysplastic cells break through the basement membrane and invade the substantia propria [118]. Since 1995, the spectrum of precancerous and cancerous epithelial lesions at the ocular surface have been united and described as OSSN [119]. The histological identification of dysplastic cells has also broadened throughout the years, and they are now recognized based on changes in cell size and shape (e.g., loss of cell polarity and increased nuclear-to-cytoplasmic ratio) and more specific nuclear deviations, such as pleomorphisms, overlap or crowding and increased number and size of nucleoli [97,120,121]. Again, pathological evaluation is not always straightforward, as some characteristics (e.g., increased nuclear-to-cytoplasmic ratio) are used to describe both atypia and dysplasia.

The thin line and overlapping characteristics to describe cellular reactions (i.e., atypia), cellular adaptations (i.e., hyperplasia and metaplasia) and preneoplastic lesions (i.e., dysplasia) complicates the molecular and histological classification of pterygium as a benign or potentially preneoplastic lesion. Based on the characteristics of dysplasia, numerous reports of unsuspected OSSN in patients with pterygium have appeared in the USA (Florida, 1% and 4.2%) [122,123], Thailand (1.8%) [124], Australia (New South Wales, 5%, mild to severe dysplasia [4]; Queensland, 9.8% [125]) and Mexico (11.29%), among others [126]. The changes observed in impression cytology that correspond to dysplasia are also of the utmost importance, as impression cytology removes the superficial layer of the ocular surface epithelia [127]. Hence, the changes should correspond to severe dysplasia. The latter, once again, emphasizes the need for a uniform classification system.

## 6. Conclusions

From a biological and cellular perspective, one would be tempted to consider pterygium as a precancerous lesion. Despite the molecular similarities, the unexpected prevalence of OSSN in pterygium tissue and the higher risk for pterygium patients to develop skin cancer [128], we are not aware of a convincing direct link between pterygium and ocular surface cancer, except for a few case reports. Moreover, the molecular progression of pterygia in untreated patients, covering several years, is unknown and awaited. Only one report includes a bilateral pterygium patient that received medical intervention after 10 years [129]. Unfortunately, molecular characterization of the biopsy is lacking.

It is also important to keep in mind that cancer is characterized by a multistep development, where cells gradually become malignant over time [130]. An important indication is that most cancers develop later in life [130]. There are proliferative disorders such as benign hemangioma that, only on very rare occasions, transform into a malignant counterpart (i.e., angiosarcoma) [131,132], while other lesions, such as gastric mucosal intestinal metaplasia, have a higher risk of resulting in gastric cancer (i.e., 0.25% of the cases) [133]. For the latter, endoscopic surveillance every 1 to 2 years is needed as follow-up [134]. Overall, the challenge to predict malignant transformation is difficult for each lesion or disorder, and often no straightforward binary criteria exist. The following questions regarding where pterygium as benign proliferative disorders with potential preneoplastic changes lies in this broad range still need to be addressed: Why is pterygium not precancerous? Is pterygium precancerous but does it harbor an additional protection mechanism to hinder its transformation towards true malignancy? Does the gap in information lie in our understanding of pterygium or in our definitions of cancerous cellular features? We hope to raise awareness regarding the use of the same histological parameters to define atypia, metaplasia and dysplasia. The importance of dysplasia and other types of OSSN in pterygium should also be considered appropriately.

In closing, despite similar UV-related etiologies and the subsequent engagement of cellular response mechanisms to neoplastic lesions, pterygium ultimately commits itself to a non-cancerous, self-limiting and superficial course. Understanding exactly why this is could help us finally unlock the nature of this ancient disease.

## Data Availability

No new data was created or analyzed in this review. Data sharing is not applicable.
